# Evaluation of the Effects of Thymoquinone on RAGE/NOX4 Expressions and Brain Tissue Morphometry in Experimental Alzheimer’s Disease Induced by Amyloid Beta 1–42 Peptide

**DOI:** 10.3390/biom15040543

**Published:** 2025-04-07

**Authors:** Şükrü Ateş, Harun Ülger, Sümeyye Uçar, Aslı Okan, Mert Ocak, Ecma Güvenilir, Zeynep Yılmaz Şükranlı, Emin Kaymak, Züleyha Doğanyiğit, Serpil Taheri, Seher Yilmaz

**Affiliations:** 1 Department of Anatomy, Faculty of Medicine, Erciyes University, Kayseri 38280, Turkey; sukru.ates@yobu.edu.tr (Ş.A.); ulger@erciyes.edu.tr (H.Ü.); sumeyyeucar@erciyes.edu.tr (S.U.); 2 Department of Anatomy, Faculty of Medicine, Yozgat Bozok University, Yozgat 66100, Turkey; 3 Department of Histology and Embryology, Faculty of Medicine, Yozgat Bozok University, Yozgat 66100, Turkey; asli.okan@bozok.edu.tr (A.O.); emin.kaymak@bozok.edu.tr (E.K.); zuleyha.doganyigit@bozok.edu.tr (Z.D.); 4 Department of Basic Medical Sciences, Anatomy, Faculty of Dentistry, Ankara University, Ankara 06100, Turkey; mocak@ankara.edu.tr; 5 Betul Ziya Eren Genome and Stem Cell Center, Erciyes University, Kayseri 38280, Turkey; 4060240047@erciyes.edu.tr (E.G.); 4021040020@erciyes.edu.tr (Z.Y.Ş.); staheri@erciyes.edu.tr (S.T.); 6 Department of Medical Biology, Faculty of Medicine, Erciyes University, Kayseri 38280, Turkey

**Keywords:** Alzheimer’s disease, thymoquinone, RAGE, NOX4, brain anatomy, micro CT

## Abstract

The onset of Alzheimer’s disease (AD) is attributed to widespread amyloid beta (Aβ) plaque accumulation, tau hyperphosphorylation, oxidative stress, and neuroinflammation. However, the underlying mechanism of AD remains unclear, and no curative treatment currently exists. The aim was to investigate the effect of thymoquinone by suppressing the RAGE/NOX4 pathway in AD. Mice (*n* = 60) were divided into five groups, and an experimental AD model induced by an Aβ_1–42_ peptide was established in two groups. We also administered 5 mg/kg thymoquinone (TMQ) to the mice for its properties to slow or treat neurodegeneration in AD. Behavioral tests for memory and emotional states, micro-computed tomography (Micro CT) to assess brain volume, ELISA to measure malondialdehyde (MDA) levels, hematoxylin and eosin staining (H&E) to evaluate neuronal degeneration were used. Immunohistochemical (IHC), Western blot (WB), and real-time polymerase chain reaction (PCR) methods were used to evaluate the inhibitory effect of TMQ on a receptor for advanced glycation end products (RAGE)/nicotinamide adenine dinucleotide phosphate (NADPH) oxidase 4 (NOX4) signaling in AD. The results showed that TMQ may have ameliorative effects on memory, spatial learning, learning ability, and anxiety in AD. We showed that TMQ has an antioxidative effect by decreasing MDA levels by the ELSIA method (*p* < 0.05). There was a marked increase in neuronal degeneration in AD mice compared to other groups (*p* < 0.05). We concluded that TMQ could ameliorate neuronal degeneration in AD by H&E staining and suppress RAGE/NOX4 signaling by IHC and WB analysis. We concluded that TMQ could be therapeutic in AD by reducing AB expression level by IHC analysis (*p* < 0.05). Real-time PCR analysis showed that *APP* (*p* < 0.05), *RAGE*, and *NOX4* (*p* < 0.05) gene expressions could be reduced by TMQ. In conclusion, TMQ has a high therapeutic potential in AD and an effective preventive and therapeutic strategy can be developed with more comprehensive studies on TMQ.

## 1. Introduction

AD, the most widespread of neurodegenerative diseases, has become a global health problem. The biggest risk factor for AD is increasing age, with the majority of patients aged 65 and over [1]. AD is marked by a gradual worsening of mental abilities. Synaptic dysfunction is the primary cause of memory dysfunction [2,3]. Alongside apathy and depression, initial clinical manifestations frequently encompass impairments in recalling recent discussions, names, or occurrences. These symptoms are the result of neurodegeneration in structures important for episodic memory, such as the hippocampus (dentate gyrus) and entorhinal cortex [4]. Neurodegeneration and synaptic dysfunctions in the hippocampus have been reported to be critical for symptoms such as memory loss and impaired cognitive function in individuals with AD [4,5].

Pathologically, disease progression is linked to extracellular amyloid beta (Aβ) plaque deposition, tau hyperphosphorylation, intracellular neurofibrillary tangles, neuroinflammation, synaptic dysfunction, and oxidative stress [6,7,8,9]. The Aβ plaque deposition hypothesis was proposed in the early 90s. According to this hypothesis, the formation of amyloid plaques or oligomers is the main factor leading to impaired synaptic transmission and neurodegeneration leading to AD [10]. Aβ_1–42_ oligomers, the main components of plaque, induce oxidative damage, increase tau hyperphosphorylation, and consequently have toxic effects on synapses and mitochondria [11,12].

The brain is vulnerable to oxidative stress due to its high oxygen demand and lipid concentration [13]. RAGE, a receptor that binds various ligands and belongs to the immunoglobulin superfamily, serves an important function in promoting neuroinflammation and oxidative stress. It is deeply implicated in the pathological processes of many diseases and is abundantly found in neurons, microglia, astrocytes, and endothelial cells in the central nervous system [7,14,15]. NOX is a key regulator of RAGE-induced reactive oxygen species (ROS) generation. Among the NOX enzyme family, NOX4 is broadly distributed in the brain and is crucial in the progression of AD as well as other neurological disorders [16,17,18]. Research results suggest that NOX4 may be a therapeutic tool based on the change in activation seen in proinflammatory genes relative to NOX4 activity [19,20,21].

Antioxidants are recognized as powerful agents in mitigating neuroinflammation and oxidative stress, both of which play central roles in the development of AD [22,23]. The therapeutic effects of black cumin seed (*Nigella sativa*) are primarily linked to its quinone compounds, with thymoquinone being the most notable among them. Thymoquinone, a potent antioxidant, is the primary bioactive compound found in the essential oil of black cumin seed [24,25]. When the antioxidant effects of thymoquinone were examined in neurodegenerative diseases, it was found to be beneficial, and considering the data, it is a substance with therapeutic potential for AD [13,26,27].

Intracerebroventricular administration of Aβ_1–42_ peptide to experimental animals such as mice and rats is one of the widely used models for the study of AD pathogenesis and effective treatments [28,29,30]. It is important to test anxiety, locomotor, learning, and memory functions in experimental animals with AD. Morris water maze, open field, and novel object recognition tests are among the frequently used tests [31,32].

As the affected brain areas increase in AD, sulci become more prominent, and the volume of gyri decreases. As a result, the morphometric volume of the brain decreases [33,34,35,36]. Imaging techniques are needed to detect these changes in the brain. Micro CT serves as the preclinical counterpart to clinical CT, offering enhanced spatial resolution for imaging disease models in small animals [37]. To achieve elevated spatial resolution, micro CT scanners employ micro-focused X-ray sources. The cross-sectional images created using X-rays are processed in the computer environment by means of appropriate software, and a 2D or 3D model of the scanned object is created in the digital environment [37,38,39].

Malondialdehyde (MDA) is the primary metabolite produced from the oxidation of cellular lipids and is regarded as a marker of lipid peroxidation [40]. In order to investigate the oxidative stress status in AD, MDA levels in the hippocampus, which is an indicator of oxidative stress, can be evaluated by ELISA test [41]. The real-time PCR technique is utilized to evaluate the expression levels of genes associated with AD, including *RAGE*, *NOX4*, *TNF-α*, *IL-1β*, and *IL-6* [42,43].

Neuronal degeneration and even neuronal death are also observed in diseases affecting the brain. H&E continues to be widely used to study neuronal degeneration in neurodegenerative diseases [37,44]. In addition, the expression of proteins associated with the disease in tissue size is tested by immunohistochemical (IHC) and Western blot (WB) analysis [44,45,46]. These methods are important in terms of revealing the pathogenesis of the disease and the mechanisms of action of the treatments applied and developing new treatment strategies [37,44,45,46].

There is no definitive conclusion on the pathogenesis and treatment of AD, and studies are currently ongoing. Consequently, the involvement of RAGE/NOX4 signaling, which is believed to contribute to oxidative stress and neuroinflammation, has not been thoroughly explored in the context of AD.

Our objective was to assess the antioxidant impact of thymoquinone on the RAGE/NOX4 signaling in a mouse model of AD induced by the Aβ_1–42_ peptide.

## 2. Materials and Methods

### 2.1. Ethical Statement

The approval of Erciyes University Experimental Animals Local Ethics Committee with the decision number 22/228 (decision date: 2 November 2022) was obtained for the compliance of the applications on mice with animal rights and animal experimentation ethics.

### 2.2. Reagents

Thymoquinone (Cat. No.: 490-91-5, Product Code: BTM-819813, Purity = 99%, Bostonchem USA Chemistry, 867 Boylston Street, Boston, MA, USA), Sesame oil (Cat. No.: HY-154629, Purity = 98.0%, MedChem Express, Monmouth Junction, NJ 08852, USA), Aβ_1–42_ peptide (Cat. No.: HY-P1388, Purity = 98.28%, MedChem Express, Monmouth Junction, NJ 08852, Monmouth Junction, NJ 08852, USA), mouse MDA (Cat. No.: BLS-8658Mo, Bostonchem USA Chemistry, 867 Boylston Street, Boston, USA) kit, Anti-Aβ (Cat. No.: E-AB-70168, Elabscience, Houston, Monmouth Junction, NJ 08852, USA), Anti-RAGE (Cat. No.: 66833-1-Ig, Proteintech, Rosemont, IL 60018, USA), Anti-NOX4 (Cat. No.: 14347-1-AP, Proteintech, Rosemont, IL 60018, USA), citrate buffer (pH: 6.0; Thermo Fischer Scientific, Cambridge CB4 0GF, UK, AP-9003-500), Ultra V block solution (Thermo Fischer Scientific, Cambridge CB4 0GF, UK, TA-125-UB), biotinylated goat anti-polyvalent secondary antibody (Thermo Fischer Scientific, Cambridge CB4 0GF, UK, TP-125-BN), streptavidin peroxidase (Thermo Fischer Scientific, Cambridge CB4 0GF, UK, TS-125-HR), diaminobenzidine (DAB) chromogen (Thermo Fischer Scientific, Cambridge CB4 0GF, UK, TA-125-HD), Gill III Hematoxylin (Merck, Darmstadt 64293, Germany, 1.05174.1000), purezol (Cat. No.: 7326890, Bio-Rad, CA 94547, USA), cDNA Synthesis Kit (Cat. No.: SRK-1000, GenetBio, Daejeon, Republic of Korea).

### 2.3. Experimental Design

A total of 60 male Balb/C mice, aged 8–10 weeks and weighing 25–30 g, were used. These mice were obtained from the Erciyes University Betül-Ziya EREN Genome and Stem Cell Center, and the experiments on mice were performed in this center.

Experimental groups:

Control group (C) (*n* = 12): No treatment was performed.

Sham group (S) (*n* = 12): Phosphate buffered Saline (PBS) was given by intracerebroventricular (i.c.v.) injection. Sesame oil was given by intraperitoneal (i.p.) injection for 14 days.

Thymoquinone group (TMQ) (*n* = 12): A total of 5 mg/kg TMQ was administered by i.p. injection for 14 days.

Alzheimer’s Disease (AD) group (*n* = 12): A total of 3 μL/5min/mouse Aβ_1–42_ peptide was administered by i.c.v. injection.

Alzheimer’s disease + Thymoquinone (AD + TMQ) group (*n* = 12): A total of 3 μL/5min/mouse Aβ_1–42_ peptide was administered by i.c.v. injection. A total of 5 mg/kg TMQ was administered by i.p. injection for 14 days after i.c.v. injection.

### 2.4. Induction of Alzheimer’s Disease and Administration of Thymoquinone

**Figure 1:**
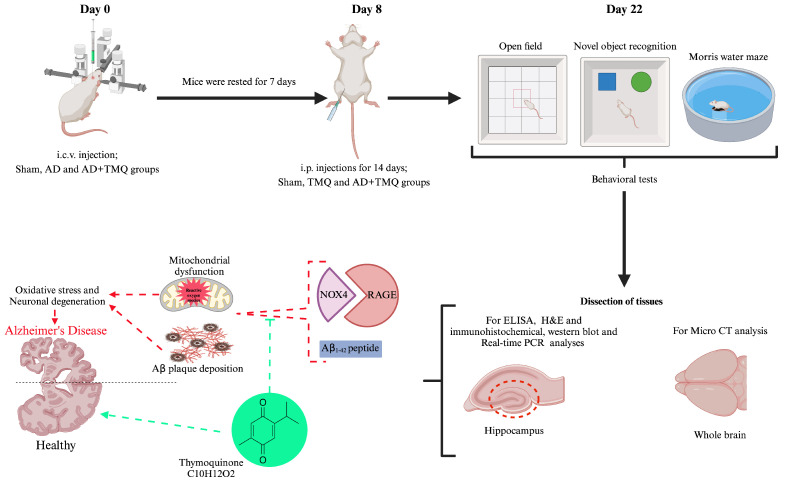
Graphical abstract of the experimental process (Biorender.com).

Aβ_1–42_ peptide was dissolved in PBS (pH 7.5, 0.1 M) at 200 μg/μL. The resulting solution was incubated at 37 °C for seven days to form fibril-like structures and oligomers. The heads of anesthetized mice were fixed with a stereotaxy device. After disinfection, a midline incision was made on the scalp from the level of the eyes to the nape of the neck, and the periosteum was scraped. A point 0.8 mm anteroposteriorly and 1.6 mm mediolaterally to Bregma was marked. The lateral ventricle of the brain was accessed with a Hamilton microsyringe through the hole drilled in the skull in the marked area and advanced to a depth of 3.5 mm. Then, Aβ_1–42_ peptide prepared in stock solution was injected i.c.v. at a dose of 3 μL/5 min/mouse. The sham group was injected with PBS i.c.v. at a dose of 3 μL/5 min/mouse [28,47]. Post-surgery, the mice were housed in individual cages with ad libitum access to food and water. The mice were rested for 7 days for recovery after i.c.v. injection. After the recovery period, the sham group was injected i.p. with sesame oil in a volume of 0.05 mL for 14 days. TMQ and AD + TMQ groups received 2.5 mg of thymoquinone dissolved in 1 mL sesame oil and injected i.p. into mice at a dose of 5 mg/kg/day in a volume of 0.05 mL for 14 days [48]. Afterwards, behavioral tests were started. After the behavioral experiments were completed, the brain tissues of the mice were removed under anesthesia. For histological tissue detection and examination, IHC analysis and micro CT analysis, brain tissues were placed whole in a bovine solution. The hippocampus was carefully dissected from brain tissues for WB, ELISA, and RNA isolation and real-time PCR analyses. The dissected hippocampus tissues were preserved at −80 °C until analysis (Figure 1).

### 2.5. Behavioral Tests

After these applications were completed, the mice were subjected to behavioral tests (open field, novel object recognition, and Morris water maze tests). The mice were brought to the environment where the tests were performed 30 min before the tests to acclimatize to the environment where the tests were to be performed.

#### 2.5.1. Open Field Test

**Figure 2:**
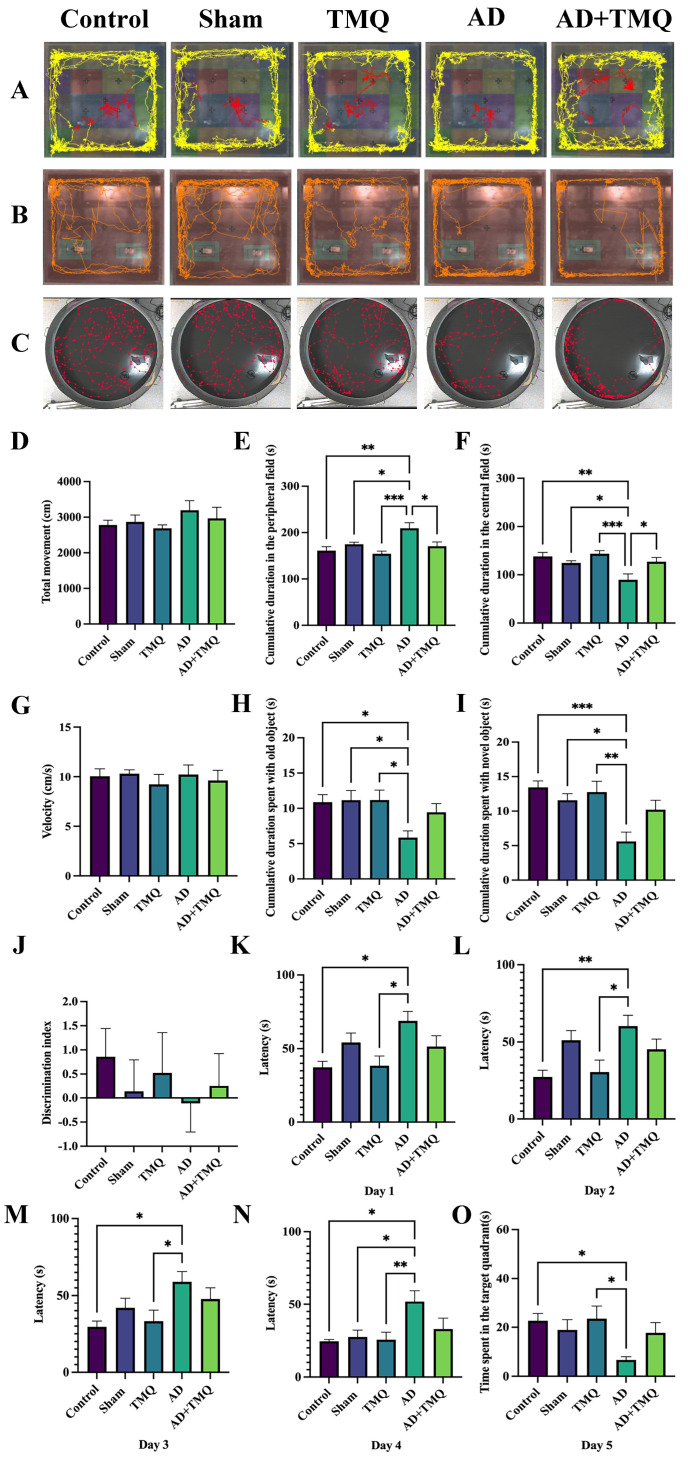
Trace visualizations of mice in the open field test (**A**), novel object recognition test (**B**), and Morris water maze test (**C**). Bar graphs showing the behavioral test results. Open field tests results: (**D**) total movement; (**E**) cumulative duration in peripheral field (s); (**F**) cumulative duration in central field (s); and (**G**) velocity (cm/s). Novel object recognition tests results: (**H**) cumulative duration spent with old object (s); (**I**) cumulative duration spent with novel object (s); and (**J**) discrimination index. Morris water maze test results: (**K**) day 1; (**L**) day 2; (**M**) day 3; (**N**) day 4; (**O**) time spent in the target quadrants (day 5). * *p* < 0.05, ** *p* < 0.01, and *** *p* < 0.001.

The test assesses different behavioral patterns, including anxiety, locomotor, exploratory, and emotional activity levels. During the test, total movement (cm), cumulative duration in the peripheral field (s), cumulative duration in the central field (s), and velocity (cm/s) are assessed. The test is performed in a black Plexiglas box (60 cm × 60 cm × 40 cm in height). Mice are positioned at the center of the box and monitored for a duration of 5 min using a video recording system. Following each trial, the box is sanitized with 70% alcohol, and after 5 min, a new mouse is introduced to the box to repeat the test. Videos were then analyzed using EthoVision XT software (Verison 9, Noldus, Wageningen, The Netherlands) (Figure 2A) [49].

#### 2.5.2. Novel Object Recognition Test

This test measures learning and memory functions. The expected behavior of mice in this experiment is to spend more time with a novel object in order to explore an object different from the one they are used to due to the novelty instinct. Two familiar objects, no larger than the mouse, were placed in the setup in which we performed the open field test. In the first stage, which consisted of two stages, the mouse was allowed to recognize the familiar objects and acclimatize to the environment for 5 min. After two hours, in the second stage, one of the familiar objects was replaced with a novel object [50]. The objects were placed on one side of the box. Mice are placed in the center of the box and recorded for 5 min with a video camera. Videos were then analyzed using EthoVision XT software (Verison 9, Noldus, Wageningen, The Netherlands) (Figure 2B). The cumulative duration (s) spent with both old (familiar) and novel objects and the discrimination index (the difference between cumulative duration (s) spent with the novel object and the cumulative duration (s) spent with the old object divided by the total time and multiplied by 100) was measured [51].

#### 2.5.3. Morris Water Maze Test

Here we assessed animals’ remote memory, near memory, and learning abilities. The water tank used for the test is usually cylindrical (50 cm in height and 120 cm in diameter). For the assessment of memory, a fixed hidden platform is placed in one quadrant of the water tank, which is divided into four quadrants. Each day for four days, on each of four consecutive trials (90 s), mice were left facing the wall of the water tank, starting from a different quadrant, and were expected to find the hidden platform. On the fifth day, the platform is removed from the water tank, and the time spent by the animals in the quadrant where the platform is located is evaluated. All these processes were recorded with a camera, and the latency (s) of the mice to find the platform in days 1–4 of the trials and the time (s) spent in the target quadrant (the quadrant where the platform was located) on the fifth day were recorded. The recordings were analyzed with EthoVision XT software (Verison 9, Noldus, Wageningen, The Netherlands) (Figure 2C) [49,52].

### 2.6. Micro CT

Brain tissues were scanned with a micro CT device (SkyScan 1275, Kontich, Belgium). Total brain volumes in mm^3^ were obtained from the scan. The configuration and parameters were as described in our previous studies [53,54].

### 2.7. ELISA Analysis

Hippocampal tissues obtained from the subjects were preserved at −80 °C. Before analysis, the tissues were homogenized on ice and subsequently subjected to centrifugation. The supernatants obtained were then collected in Eppendorf tubes for further examination. The concentration of malondialdehyde (MDA) was quantified using an ELISA method with a mouse MDA kit, measured in pg/mL at 450 nm using the BMG LABTECH Spectrostar Nano device [55].

### 2.8. Hematoxylin and Eosin Staining

Whole brain tissues were first fixed in a 10% formaldehyde solution. Following this step, they were rinsed in running water overnight. Subsequently, they underwent a dehydration process by passing through a graded series of alcohol concentrations (70%, 80%, 96%, and 100%). The tissues were then cleared with xylene and embedded in fresh paraffin.

H&E staining was performed on 5 μm thick sections taken from paraffin blocks, and histopathological changes (neuronal degeneration, vascular dilatation) [56] were examined under a light microscope (Zeiss Axioscope 5, Carl Zeiss Meditec AG, 73447 Oberkochen, Germany) and imaged with a digital camera (Zeiss Axiscope Colibri 3, Carl Zeiss Meditec AG, 73447 Oberkochen, Germany). Neuronal degeneration was determined with reference to Tampe et al., 2016 [57]. Histopathologic results were scored according to Doğanyiğit et al., 2023 [45]. Quantification was performed randomly and blindly by two researchers. Histomorphologic scoring was performed for a minimum of 10 images per mice, for at least 3 mice per experimental group.

### 2.9. Immunohistochemical Analysis

IHC staining for Aβ, RAGE, and NOX4 was performed by avidin–biotin peroxidase method in total brain tissue sections obtained from the experimental groups [58]. Sections were prepared for incubation with primary antibodies according to the procedure used by Doğanyiğit et al., 2023 [45]. Then incubated with primary antibody (Amyloid beta 1:200, RAGE 1:200, and NOX4 1:200 dilution ratios) overnight at 4 °C. Then incubated with biotinylated goat anti-polyvalent secondary antibody for 40 min at room temperature. After several washes with PBS, they were incubated with streptavidin peroxidase for 30 min at room temperature. The antibody complex was visualized by incubation with diaminobenzidine (DAB) chromogen. Sections were then counterstained with Gill III Hematoxylin. Sections were dehydrated by passing through the remaining alcohol series and covered with entellan. Sections were examined and visualized with a ZEISS Axiscope 5 (Carl Zeiss Meditec AG, 73447 Oberkochen, Germany) light microscope and imaged with a digital camera (Zeiss Axiscope Colibri 3, Carl Zeiss Meditec AG, 73447 Oberkochen, Germany). Immunoreactivity levels were measured in the ImageJ software (1.46, NIH, Bethesda, MD, USA) program.

### 2.10. Western Blot Analysis

WB analysis was performed for semiquantitative evaluation of RAGE, and NOX4 proteins in hippocampus tissue samples obtained from the experimental groups. Protein content of hippocampus tissue samples was determined according to Okan et al., 2024 [58]. From each hippocampus sample, 100 micrograms of protein was loaded onto 8% SDS-PAGE. After electrophoresis, proteins were transferred to polyvinylidene difluoride (PVDF) membranes (Bio-Rad). To reduce non-specific binding, the membranes were blocked with 5% bovine serum albumin (BSA) in TBS-T buffer (0.1% Tween-20, Tris-buffered saline) for 1 h. The membranes were then incubated with the primary antibody (1/1000 dilution ratio) for 1.5 h at room temperature. Beta-actin was used as an internal control. The membranes were then washed with TBS-T 3 times for 10 min each and incubated with peroxidase-labeled goat-anti-rabbit IgG (Abcam, Cambridge CB2 0AX, UK) for 1 h at room temperature. For visualization, the chemiluminescence-based SuperSignal CL-HRP Substrate System (Thermo Scientific, Waltham, MA, USA) was used according to the manufacturer’s instructions, and then band images were captured with a Syngene GeneGnome XRQ (Syngene, Cambridge, CB4 1TF, UK) instrument. Band images were quantified in the ImageJ (1.46, NIH, Bethesda, MD, USA) software program, and the expression of proteins was determined by proportioning with beta-actin band images [59].

### 2.11. RNA Isolation and Real-Time PCR Analysis

Hippocampus samples were homogenized by adding 500 μL of Purezol to the Eppendorf tubes containing the samples. RNA extraction was then carried out according to the protocol provided by the manufacturer, and complementary DNA (cDNA) was generated from the isolated RNA samples using the SuPrimeScript cDNA Synthesis Kit. cDNA preparation followed the instructions outlined by the manufacturer, and sample quantification was conducted on a LightCycler 480 II system (Roche Diagnostics Ltd., Rotkreuz, Switzerland). The expression levels of *APP*, *RAGE*, and *NOX4* genes in the hippocampus across all groups were assessed following their respective methodologies. The beta-actin gene served as the reference gene. Each PCR experiment was conducted in duplicate, and Ct values were standardized using the 2^−ΔΔCt^ method for normalization [60]. The sequences of the primers are shown in Table 1.

### 2.12. Statistical Analysis

Statistical analyses were conducted using the GraphPad Prism 9 (Version 9.5.0) software. The Shapiro–Wilk test was applied to assess the normal distribution of the data. Numerical results from behavioral tests, micro CT, ELISA, H&E, IHC, and real-time PCR analyses were compared using one-way ANOVA and followed by Tukey’s multiple comparison test (*F* (DFn; degrees of freedom between groups, DFd; degrees of freedom within groups) = *F* value, *p* = *p* value). For data that did not follow a normal distribution, the Kruskal-Wallis test with multiple comparisons was utilized (*H*(df; number of groups − 1) = *H* value, *p* = *p* value). Data from the Western blot (WB) analysis were compared with the control group using one-way ANOVA and Dunnett’s multiple comparison test. The results of all data analyses are shown as bar graphs. The data in the bar graphs are expressed as mean ± standard error mean (SEM) and *p* < 0.05 was accepted as a statistically significant difference.

## 3. Results

### 3.1. Behavioral Tests Results

#### 3.1.1. Open Field Test Results

In this test in which behavioral patterns such as anxiety, locomotor, and emotional activity were analyzed, the cumulative duration (s) and velocity (cm/s) data of the mice in total movement (cm), peripheral, and central fields were evaluated. It was observed that there was an increase in total movement in the AD group mice, but there was no statistically significant difference between the groups (*F* (4, 35) = 0.804, *p* = 0.530) (Figure 2D). When the data in cumulative duration (s) in the peripheral field were analyzed, it was seen that the data of the AD group were higher than the control, sham, TMQ, and AD + TMQ groups, and the difference was statistically significant (*F* (4, 35) = 6.344, *p* = 0.0006) (Figure 2E). When the cumulative duration (s) in the central field was analyzed, it was seen that the data belonging to the AD group were lower than the control, sham, TMQ, and AD + TMQ groups, and the difference was statistically significant (*F* (4, 35) = 6.257, *p* = 0.0007) (Figure 2F). When the velocity (cm/s) data were evaluated, there was no difference between the groups (*F* (4, 35) = 0.276, *p* = 0.891) (Figure 2G).

#### 3.1.2. Novel Object Recognition Test Results

In this test in which learning and memory functions were measured, the cumulative duration (s) spent with old and novel objects and discrimination indices were evaluated. The cumulative duration (s) spent with both old (*F* (4, 35) = 3.492, *p* = 0.016) and novel (*F* (4, 35) = 6.071, *p* = 0.0008) objects was lower in the AD group compared to the control, sham, and TMQ groups, and the difference was statistically significant. Although the cumulative duration (s) spent with old and novel objects was lower in the AD group compared to the AD + TMQ group, the difference was not statistically significant (*p* > 0.05) (Figure 2H,I). In the discrimination index data, the AD group had the lowest value, but the difference between the groups was not statistically significant (*F* (4, 35) = 0.304, *p* = 0.872) (Figure 2J).

#### 3.1.3. Morris Water Maze Test Results

Days 1–4 is the phase in which the location of the platform is learned. It was observed that the latency (s) time decreased as the days progressed. When the data were evaluated, it was observed that the latency to find the platform was longer in the AD group compared to the control and TMQ groups in all four days, and the difference was statistically significant (Day 1, *H* (4) = 13.07, *p* = 0.010; Day 2, *H* (4) = 18.39, *p* = 0.001; Day 3, *H* (4) = 14.71, *p* = 0.005) (Figure 2K–N). On day 4, the latency of the sham group to find the platform was less than the AD group, and the difference was statistically significant (*H* (4) = 14.65, *p* = 0.005) (Figure 2N).

On the fifth day of the test, the platform in the tank was removed. The time spent by the mice in the quadrant where the platform was located was evaluated. Since the mice had learned the location of the platform, they were expected to spend more time in the target quadrant to find the platform. When the data were evaluated, it was seen that the time spent in the target quadrant was less in the AD group compared to the control and TMQ groups, and the difference was statistically significant (*F* (4, 25) = 3.122, *p* = 0.032) (Figure 2O).

### 3.2. Micro CT Results

**Figure 3:**
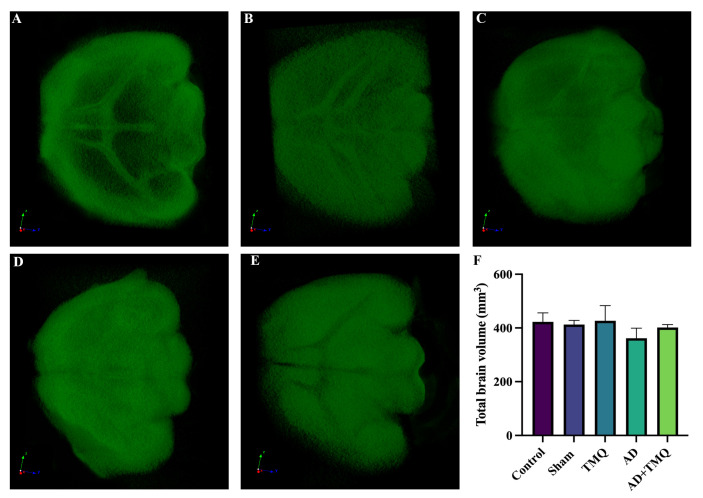
Micro CT images of brain tissue of mice (**A**–**E**). (**A**) Control; (**B**) Sham; (**C**) TMQ; (**D**) AD; (**E**) AD + TMQ groups. Bar graph (**F**) showing the total brain volume (mm^3^).

Micro CT images of brain tissues are shown in Figure 3A–E. When total brain volume measurements were evaluated, a reduction in volume was noted in the AD group when compared to the other groups. Nevertheless, there were no statistically significant differences in volume measurements among the groups (*F* (4, 10) = 1.669, *p* = 0.232) (Figure 3F).

### 3.3. ELISA Results

**Figure 4:**
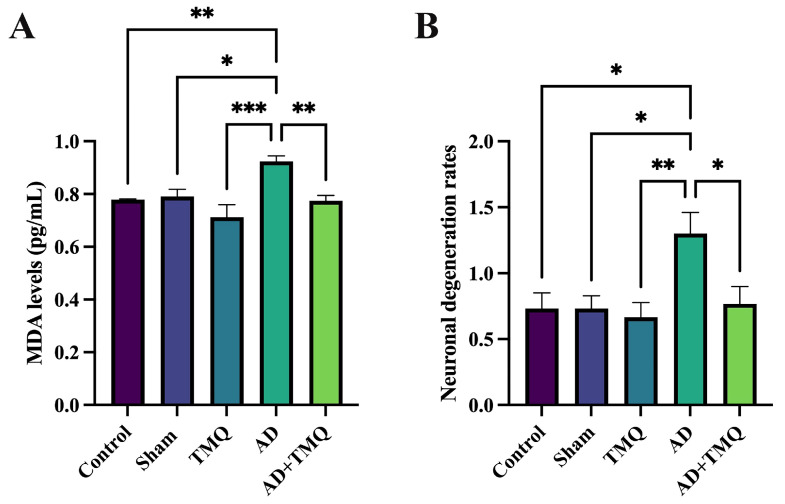
MDA levels (pg/mL) in hippocampus tissue (**A**). Neuronal degeneration rates in hippocampus (**B**). * *p* < 0.05, ** *p* < 0.01, and *** *p* < 0.001.

With this method, MDA levels in hippocampus tissues, which are expected to increase with oxidative stress, were analyzed. When the data were evaluated, the MDA level in the AD group was elevated compared to that in the control group (*F* (4, 25) = 7.834, *p* = 0.0003). No statistically significant differences were found among the control, sham, TMQ, and AD + TMQ groups (*p* > 0.05) (Figure 4A).

### 3.4. Histological Analysis

**Figure 5:**
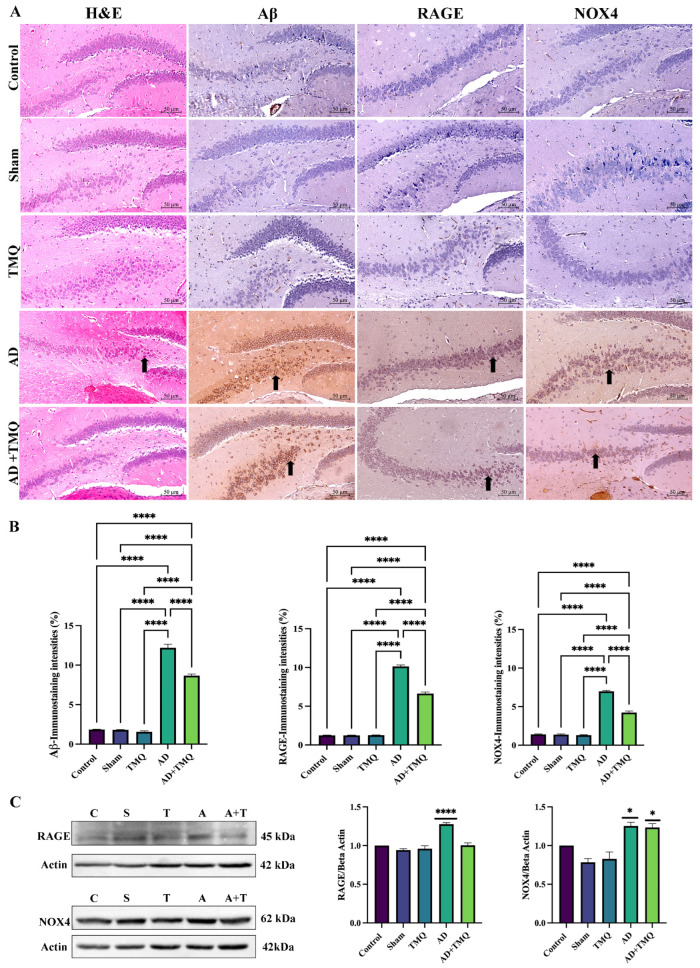
H&E staining and Aβ, RAGE, and NOX4 IHC staining images in the hippocampus of the experimental groups. Magnification 20× and scale bar 50 μm (**A**). The black arrows indicates neuronal degeneration in H&E staining, while IHC staining shows protein expression in the stained neurons. Bar graphs showing the immunostaining intensities (**B**). Band images obtained by WB analysis in hippocampus tissue samples and bar graphs showing RAGE and NOX4 protein expression relative to internal control beta-actin (**C**). C, Control; S, Sham; T, TMQ; A, AD; A + T, AD + TMQ (**C**). * *p* < 0.05 and **** *p* < 0.0001. (Original Western Blot figures see Supplementary Materials).

Histopathologic changes in the hippocampus areas of the brain of the mice in the experimental groups were examined using a light microscope after hematoxylin and eosin staining (Figure 4B and Figure 5A). No differences in vascular dilatation were observed among the groups (*F* (4, 144) = 1.856, *p* = 0.121). Neuronal degeneration in the AD group was higher than in control, sham, TMQ and AD + TMQ groups. In the AD + TMQ group, neuronal degeneration was less than in the AD group (*F* (4, 145) = 4.310, *p* = 0.002) (Figure 4B).

### 3.5. Immunohistochemical Analysis Results

As shown in Figure 5A, when Aβ, RAGE, and NOX4 expressions were analyzed in hippocampus samples, it was observed that the immunoreactivity levels observed in the AD group showed a significant increase in comparison to the control, sham, TMQ and AD + TMQ groups. In the AD + TMQ group, the immunoreactivity levels of the antibodies examined decreased statistically significantly in comparison to the AD group (Aβ: *F* (4, 25) = 461.9, *p* < 0.0001; RAGE: *F* (4, 25) = 1096, *p* < 0.0001; NOX4: *F* (4, 25) = 432.3, *p* < 0.0001)) (Figure 5B).

### 3.6. Western Blot Analysis Results

In hippocampus samples (Figure 5C), RAGE protein expression was increased in the AD group compared to the control group (*F* (4, 10) = 31.04, *p* < 0.0001). When NOX4 expression was analyzed, it was found to be significantly increased in both the AD and AD + TMQ groups compared to the control group (*F* (4, 10) = 16.20, *p* = 0.0002) (Figure 5C).

### 3.7. Real-Time PCR Results

**Figure 6:**
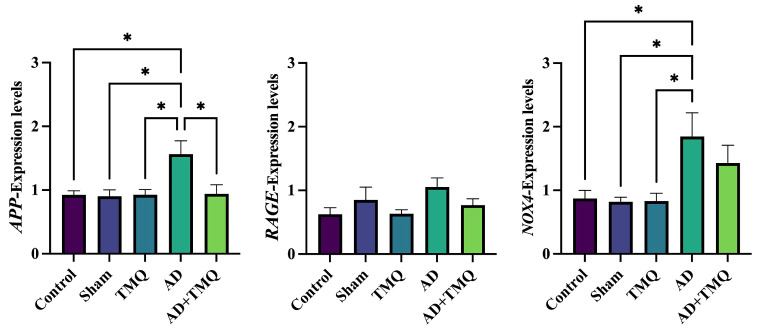
Bar graphs showing the expression levels of *APP*, *RAGE*, and *NOX4* genes as a result of real-time PCR analysis in hippocampus tissue samples. * *p* < 0.05.

According to the results obtained, *APP* gene expression level was markedly elevated in the AD group comparison to the control, sham, TMQ, and AD + TMQ groups in hippocampus tissue samples (*F* (4, 20) = 4.807, *p* = 0.007). *RAGE* gene expression reached its highest level in the AD group; however, there was no statistically significant difference among the groups (*F* (4, 20) = 1.835, *p* = 0.161). *NOX4* gene expression level was the highest in the AD group, and the difference between the control, sham, and TMQ groups and the AD group was statistically significant (*F* (4, 20) = 4.212, *p* = 0.012). *NOX4* gene expression level was lower in the AD + TMQ group compared to the AD group, but the difference was not statistically significant (*p* > 0.05) (Figure 6).

## 4. Discussion

Neuronal degeneration and synaptic dysfunctions in the hippocampus are responsible for impaired memory and cognitive functions [4,5]. The Aβ peptide (especially Aβ_1–42_), which is most commonly responsible for amyloid plaques, neurofibrillary tangles, synapse loss, and neuronal degeneration, has been implicated as a target in AD pathogenesis. It is hypothesized that the aggregation of Aβ plaques is succeeded by the hyperphosphorylation of tau protein [61,62,63]. Another factor responsible for AD pathogenesis is oxidative stress. The brain’s elevated oxygen demand and substantial lipid composition, combined with its limited antioxidant defenses, render it particularly susceptible to oxidative stress [13,64].

Thymoquinone (2-isopropyl-A5-methylbenzo-1,4-quinone), a potential chemical constituent of Nigella Sativa, an annual herbaceous plant belonging to the Ranunculaceae family, is reported to have a neuroprotective effect [26,65]. In vivo studies have reported neuroprotective effects of thymoquinone when administered at doses of 5 mg/kg [66,67]. In the open field test, the amount of time rats spend in the peripheral or central field is correlated with anxiety [68]. In an experimental AD mouse model, a significant reduction of entries into the central area and time spent in the central region of the open field was observed in AD mice in the open field test [69]. In a study on APP/PS1 mice in which ethanol exposure altered Alzheimer’s-related pathology, it was reported that mice exposed to ethanol spent less time in the central area [70]. In this study, it was observed that AD group mice exhibited anxiety-related behaviors by spending more time in the peripheral area, and TMQ administration suppressed these behaviors.

In a study conducted on double-transgenic AD mice (APPswe/PSEN1dE9), known as APP/PS1 mice, it was reported that the number of novel object explorations and the discrimination index of the mice decreased [71]. In another study, it was observed that the novel object exploration time and discrimination index of the mice in the AD model created in Aβ_1–42_-induced male ICR mice were low [72]. In another study conducted on the Aβ_1–42_-induced AD model in *Balb/C* mice, it was reported that the discrimination index of AD mice was low [73]. In our study, mice were subjected to training and testing for 5 min. In different studies, it was observed that these durations were 10 min, and there were also two training phases [74,75]. Looking at the data obtained by Gök et al. (2024) similar to our data, the cumulative time spent by mice with objects was below 15 s [51]. The time spent on objects and discrimination index values of mice with AD were lower compared to other groups. Although the data of the AD + TMQ group were slightly more promising, there was no statistical difference between the AD groups. We think that the use of modified versions of the test by different researchers has led to possiblly different results.

In Aβ_1–42_-induced experimental AD in rats, TMQ treatment restored hippocampal neuron loss. In Morris water maze test results, TMQ treatment also improved spatial memory in AD rats [76]. Different studies have also shown that TMQ-treated experimental animals performed better in the Morris water maze test [67,77]. Our findings from the Morris water maze test results support the findings of other studies and suggest that TMQ may prevent memory and spatial learning loss.

It has been reported that there may be a loss of brain volume as a result of neuronal degeneration [35,36]. According to our micro CT results, there was a volumetric loss in the AD group mice. The brain volume of the AD + TMQ group was higher than the AD group, but no significant difference was observed between the groups. Malondialdehyde (MDA), which is considered an index of lipid peroxidation [40], has been reported to be at high levels in the hippocampus tissue in AD [41]. In studies using an in vivo AD model, it was emphasized that thymoquinone exhibited antioxidant properties by reducing MDA levels in brain tissue [78,79]. Our ELISA results showed that TMQ decreased the level of MDA, a marker of oxidative stress, in mice with AD.

RAGE/NOX4 is an effective signaling pathway on oxidative stress. With the idea that NOX4 contributes to RAGE-mediated ROS production in the progression of AD, its role in AD pathogenesis is being investigated, and it is thought to be a therapeutic target for the treatment of the disease [14,17,18,21]. In the Aβ_1–42_-mediated AD mice model, the expression level of RAGE in the hippocampus was reported to be significantly increased in the AD model group as a result of IHC and WB analysis [72]. In another study, it was reported that RAGE expression level was high in AD mouse brains using WB analysis [80]. In the AlCl3-induced AD model on C57BL/6 mice, RAGE and NOX4 expression levels were observed to be increased in the brain tissues of AD mice as a result of WB blot analysis [7]. Another study reported that Aβ and NOX4 expressions in the hippocampi of AD mice were significantly increased [17]. There are very few studies in the literature examining the effects of thymoquinone on RAGE and NOX4 in AD. In the present study, the results were supportive of previous studies, and it was shown that TMQ can reduce Aβ, RAGE, and NOX4 expressions in hippocampus samples of AD mice.

## 5. Conclusions

In conclusion, it is seen that our study has information supporting amyloid beta and oxidative stress hypotheses in AD pathogenesis. In line with the information in the literature, data supporting the hypothesis that RAGE/NOX4 may be a therapeutic target in AD were obtained. Accordingly, it has been shown that TMQ may have an antioxidative potential by suppressing RAGE/NOX4 expressions and reducing MDA levels and may be effective in reducing neuronal degeneration. Micro CT can be alternative imaging method for AD. It is important to clarify the relationship between RAGE/NOX4 and AD and further detailed studies are needed in the future. Antioxidants such as thymoquinone may be effective in AD but this needs further investigation.

## Figures and Tables

**Table 1:** Primers’ forward (F) and reverse (R) sequences.

Primer (Gen) Name	Primers’ Sequences
*APP*-F	GTCGCCAAAGAGACATGCAG
*APP*-R	CCCCTCG GAACTTGTCGATG
*RAGE*-F	CCGAGTCCGAGTCTACCAGA
*RAGE*-R	CGCAGTGTAAAGAGTCCCGT
*NOX4*-F	TCACCCTCGCTGCATTA
*NOX4*-R	ACTTGGGTTCTTCCAGGCAAA

## Data Availability

The data presented in this study are available from the corresponding author upon request.

## Supplementary Materials

The following supporting information can be downloaded at: https://www.mdpi.com/article/10.3390/biom15040543/s1, Figure S1: Original Western Blot Images.

## Abbreviations

The following abbreviations are used in this manuscript:

AD	Alzheimer’s disease
Aβ	amyloid beta
*APP*	amyloid beta precursor protein
Micro CT	micro-computed tomography
MDA	malondialdehyde
H&E	hematoxylin and eosin
IHC	immunohistochemical
WB	Western blot
PCR	polymerase chain reaction
TMQ	thymoquinone
RAGE	receptor for advanced glycation end products
NADPH	nicotinamide adenine dinucleotide phosphate
NOX4	NADPH oxidase 4.
