# Supplementary figures and images for: Evaluation of the Effects of Thymoquinone on RAGE/NOX4 Expressions and Brain Tissue Morphometry in Experimental Alzheimer’s Disease Induced by Amyloid Beta 1–42 Peptide
Source: Biomolecules. 2025 Apr 7;15(4):543. doi: 10.3390/biom15040543 (PMC12024666; doi:10.3390/biom15040543)

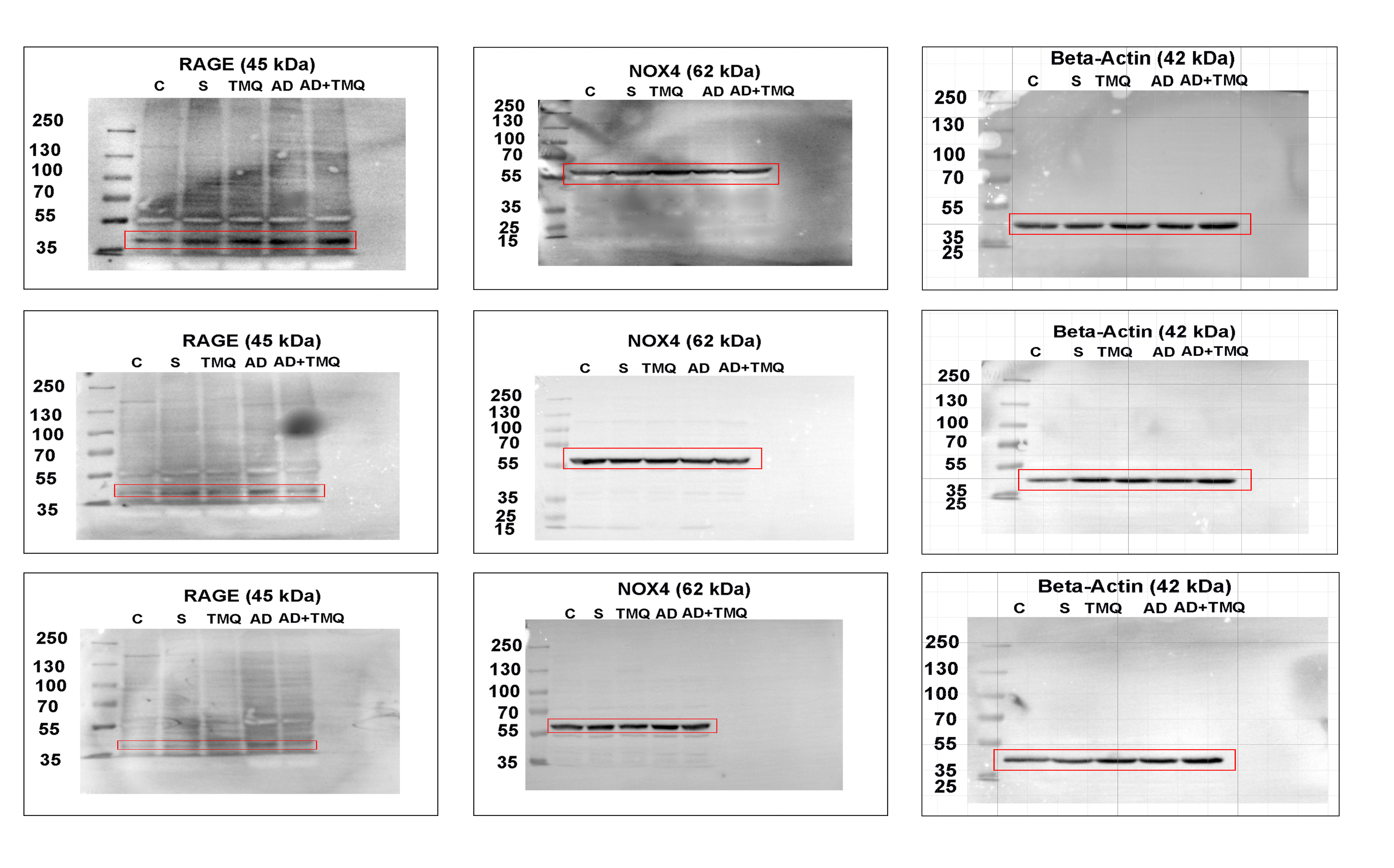

Supplement: Supplementary file 1 [file biomolecules-15-00543-s001.zip › Figure S1. WB-original blots.tif]
